# Predictors of SARS-CoV-2 Infection in Youth at a Large, Urban Healthcare Center in California, March–September 2020

**DOI:** 10.3389/fped.2021.752247

**Published:** 2021-11-17

**Authors:** Caitlin N. Newhouse, Tawny Saleh, Trevon Fuller, Tara Kerin, Mary C. Cambou, Emma J. Swayze, Catherine Le, Wonjae Seo, Marisol Trejo, Omai B. Garner, Sukantha Chandrasekaran, Karin Nielsen-Saines

**Affiliations:** ^1^Department of Medicine, Preventive Medicine Program, David Geffen School of Medicine at the University of California, Los Angeles, Los Angeles, CA, United States; ^2^Department of Pediatrics, Division of Pediatric Infectious Diseases, David Geffen School of Medicine at the University of California, Los Angeles, Los Angeles, CA, United States; ^3^Institute of the Environment & Sustainability, University of California, Los Angeles, Los Angeles, CA, United States; ^4^Laboratorio de Doenças Febris Agudas, Instituto Nacional de Infectologia, Fundação Oswaldo Cruz, Rio de Janeiro, Brazil; ^5^Department of Medicine, Division of Infectious Diseases, David Geffen School of Medicine at the University of California, Los Angeles, Los Angeles, CA, United States; ^6^Western Michigan University Homer Stryker M.D. School of Medicine, Kalamazoo, MI, United States; ^7^Department of Pathology and Laboratory Medicine, David Geffen School of Medicine at the University of California, Los Angeles, Los Angeles, CA, United States

**Keywords:** COVID-19, SARS-CoV-2, youth, testing, viral load, LA County

## Abstract

**Objective:** To understand which social, epidemiologic, and clinical risk factors are associated with SARS-CoV-2 infection in youth accessing care in a large, urban academic institution.

**Methods:** We conducted a prospective cohort study with case–control analyses in youth who received testing for SARS-CoV-2 at our academic institution in Los Angeles during the first wave of the COVID-19 pandemic (March–September 2020).

**Results:** A total of 27,976 SARS-CoV-2 assays among 11,922 youth aged 0–24 years were performed, including 475 youth with positive SARS-CoV-2 results. Positivity rate was higher among older, African American, and Hispanic/Latinx youth. Cases were more likely to be from non-English-speaking households and have safety-net insurance. Zip codes with higher proportion of Hispanic/Latinx and residents living under the poverty line were associated with increased SARS-CoV-2 cases. Youth were more likely to have positive results if tested for exposure (OR 21.5, 95% CI 14.6–32.1) or recent travel (OR 1.5, 95% CI 1.0–2.3). Students were less likely to have positive results than essential worker youth (OR 0.5, 95% CI 0.3–0.8). Patterns of symptom presentation varied significantly by age group; number of symptoms correlated significantly with age in SARS-CoV-2 cases (*r* = 0.030, *p* < 0.001). SARS-CoV-2 viral load did not vary by symptom severity, but asymptomatic youth had lower median viral load than those with symptoms (21.5 vs. 26.7, *p* = 0.009).

**Conclusions:** Socioeconomic factors are important drivers of SARS-CoV-2 infection in youth. Presence of symptoms, exposure, and travel can be used to drive testing in older youth. Policies for school reopening and infection prevention should be tailored differently for elementary schools and universities.

## Introduction

In the early weeks of the Coronavirus Disease (COVID-19) pandemic, severe acute respiratory syndrome coronavirus-2 (SARS-CoV-2) testing was limited to those with high risk for development of severe disease or known epidemiologic risk factors (1). Despite improved testing capacity, testing volume has remained lower in youth than adult populations (2). This resulted in a scarcity of data to guide public health and clinical decisions about SARS-CoV-2 testing and infection prevention practices in youth populations.

While the symptoms of COVID-19 have been shown to overlap with other common viral respiratory infections, the spectrum of COVID-19 disease in youth across age groups has not been well-established (3–5). Prior studies suggested that symptoms may not be reliable to direct clinical decision-making around testing (4, 6); therefore, identifying epidemiologic risk factors for positive SARS-CoV-2 tests could inform testing strategies for clinicians and infection prevention protocols. Studies have shown that many children who test positive for SARS-CoV-2 have close contact with a parent or sibling who have symptoms or confirmed infection (7–9). Additionally, socioeconomic inequality, including place of residence, has been established as a driver of SARS-CoV-2 infection in adult populations (10–12), and race, as a proxy for systemic racism, has been shown to be a risk factor for infection in youth (3, 7, 13, 14). However, to our knowledge, geographic epidemic modeling of children and youth in southern California are not yet available. Finally, viral kinetics, including viral load and duration of shedding have been described in adult and mixed adult and pediatric cohorts, but no studies have been done in a large population of American youth (15–18).

To our knowledge, the direct comparison of these social, epidemiologic, and clinical risk factors for SARS-CoV-2 infection, including symptoms, exposure, occupation, and geographical location in conjunction with viral kinetics has not been done before in an American youth population. We undertook this study to better understand which social, epidemiologic, and clinical risk factors are associated with SARS-CoV-2 infection in youth presenting to care in a large academic Medical Center in Southern California, a diverse and geographically widespread area, with a special focus in Los Angeles County. Especially as we move into the second year of the COVID-19 pandemic, it is essential to understand the disease dynamics among youth, many of whom are not yet eligible for COVID-19 vaccination in most of the world.

## Materials and Methods

### Population, Setting, and Data Collection

We conducted a prospective cohort study of a convenience sample of youth (age <25 years) presenting to a large academic medical center (Ronald Reagan Medical Center and Mattel Children's Hospital at UCLA), a community hospital (UCLA Santa Monica Hospital), and a widespread network of UCLA-affiliated clinics in Los Angeles County from March through September 2020. Inclusion in the cohort was performed through an Institutional COVID-19 REDCap Registry (19). De-identified data were abstracted by our Institutional COVID-19 Data Registry controlled by our institution's Clinical and Translational Science Institute (CTSI) Biostatistics Unit. All patients received testing for SARS-COV-2 *via* reverse-transcriptase polymerase chain reaction (RT-PCR) or serology. Confirmed cases of COVID-19 were defined as any positive RT-PCR or serologic assay result. If a patient had multiple tests done, they were considered a confirmed case if at least one serologic or RT-PCR test was positive. For our case–control analysis, age-matched negative controls were selected randomly from all patients <25 years of age with negative RT-PCR or serology for SARS-CoV-2.

Basic demographic data (age, gender, race/ethnicity, and zip code) were provided by our Institutional COVID-19 Registry on all patients in the cohort. Detailed chart abstraction was performed by the CTSI Biostatistics Unit including members of the study team on confirmed cases and age-matched negative controls. A positive IgG serology result was interpreted as evidence of prior infection with COVID-19. Sex, past medical history, and insurance type were identified as potential confounding variables. Study activities were approved by the UCLA Institutional Review Board that provided an IRB exemption.

### Primary and Secondary Variables

Our primary outcome of interest was a positive RT-PCR or serology. Our primary exposure of interest was zip code of the primary place of residence. Our secondary exposure of interest was reason for testing, obtained from order placed for SARS-CoV-2 test and chart review. Reason for testing was operationalized as (1) asymptomatic surveillance, defined as received testing for screening without symptoms concerning for COVID-19 prior to an inpatient or outpatient procedure, hospital admission, or for school or travel; (2) symptoms, defined as received testing due to symptoms that were concerning for possible COVID-19; (3) exposure, defined as received testing due to known exposure to a close contact with known or suspected COVID-19; or (4) other.

Other secondary variables included race/ethnicity, household size, symptoms at presentation for testing, gender, insurance type, and parent/patient occupation. All secondary variables were obtained *via* chart review. Household size was defined as the number of people residing at the same residential address as the patient, including the patient. Language spoken at home was operationalized as English or Non-English. Insurance type was categorized as (1) MediCal/Safety Net, (2) Private (including Health Maintenance Organization and Preferred Provider Organization), and (3) None. Past medical history was identified from ICD-10 codes and medical diagnoses listed in medical notes and included obesity (defined as BMI > 98th percentile), asthma, chronic pulmonary disease, cardiac disease (chronic cardiac disease, arrythmia, myocardial infarction, or congestive heart failure), immunosuppression (including rheumatologic disorder, active cancer, HIV, or asplenia), other, and none (unknown past medical history or previously healthy).

All confirmed cases were categorized for COVID-19 disease severity according to an adaptation of the NIH Severity of Illness Categories for COVID-19 Disease for Adults (20). Asymptomatic was defined as having no symptoms consistent with COVID-19. Mild was defined as individuals with any signs and symptoms of COVID-19 (including fever, chills, headache, cough, etc.) without shortness of breath, dyspnea, or abnormal chest imaging. Moderate was defined as evidence of lower respiratory disease in clinical notes (shortness of breath or dyspnea) or abnormal chest imaging in the setting of oxygen saturation at least ≥94% on room air. Severe was defined as oxygen saturation <94% on room air, a ratio of arterial partial pressure of oxygen to fraction of inspired oxygen (PaO_2_/FiO_2_) <300 mmHg, tachypnea with respiratory frequency >30 breaths per min, or chest imaging with lung infiltrates notes in >50% lung fields. Critical was defined as individuals with respiratory failure, septic shock, and/or multiorgan failure or dysfunction. Patients with a diagnosis of multisystem inflammatory syndrome in children (MIS-C) were also categorized.

Symptoms and vital signs at the time of testing visit were included in a secondary analysis. Patients who had a hospital admission associated with SARS-CoV-2 testing were included in the outcome analysis. Outcomes included the following: hospitalization (yes/no), hospital length of stay, admission to intensive care unit (ICU), mechanical ventilation (yes/no), and death.

### Laboratory Testing

The UCLA Clinical Microbiology Laboratory performed SARS-CoV-2 RT-PCR testing. Nasopharyngeal, oropharyngeal, and nasal swabs were analyzed on one of four platforms: (1) MIC Focus 3M Integrated Cycler Analyzer (Focus Diagnostics, Diasorin Group, Saluggia, Italy), which targets the ORF1ab and S genes; (2) MIC ABI 7500 PCR System Analyzer (Bio-Rad, Hercules, CA), which targets ORF1ab, N, and S genes; (3) MIC BD Max PCR Analyzer 2 (BD Max Systems, Becton, Dickinson and Company, Franklin Lakes, NJ), which targets N1, N2, and S genes; and (4) MIC Roche 6800 (Roche Diagnostics, Indianapolis, IN), which targets ORF1ab and E genes. Send-out laboratory assays were reported through either the Happy Together Laboratory app or from Quest Diagnostics (Secaucus, NJ) to our microbiology laboratory. Ct values were not reported for outside facilities. Serologic testing in the UCLA microbiology laboratory was performed *via* enzyme-linked immunosorbent assay (ELISA) (Diasorin, Saluggia, Italy) with the target of spike receptor-binding protein IgG.

Cycle threshold (Ct) values were analyzed for all positive RT-PCR tests; positive RT-PCR was defined as Ct <40. For each patient with a positive result RT-PCR, the assay used and Ct values for each target were recorded. For patients with more than one target positive, the lowest Ct value > 0 was used. Ct value was interpreted as inversely related to viral load. An in-house analysis found comparable Ct ranges without significant differences across the four assays, suggesting that semi-quantitative grouping was acceptable across our in-house PCR assays (21).

### Data Analysis

Descriptive analysis of the demographic and testing characteristics for all patients <25 years of age with testing results in our health system was performed. We compared the percentage of participants who tested positive among different age groups and by race/ethnicity using ANOVA followed by Tukey's *post-hoc* test. Maps describing the reach, positivity rate, and absolute number of positive SARS-CoV-2 tests done at UCLA were created with ArcMap 10.6.1. Previous work has posited a relationship between race/ethnicity and SARS-CoV-2 risk due to differences in occupational exposure and use of public transportation. We modeled the number of SARS-CoV-2 cases per zip code as a function of demographic variables, economic variables, and sampling effort. The data set comprised samples from 194 zip codes. We obtained the race/ethnicity for the period 2014–2019 from the American Community Survey of the US Census Bureau (22). Race/ethnicity was highly correlated with economic and language-based variables. For example, the proportion of families living in poverty per zip code was 60% correlated with the proportion of Hispanic/Latino residents, and the proportion of residents who primarily spoke a language other than English in the home was 90% correlated with the proportion of Hispanic/Latinx residents. As language other than English and families in poverty were highly correlated with the proportion of the population that was Hispanic/Latinx, we only included the ethnicity variable in the model. The statistical model represented the number of SARS-CoV-2 cases per zip code as a Poisson distributed random variable. In the model, the number of SARS-CoV-2 cases per zip code depended on the racial/ethnic composition of the zip code and the total number of samples that were tested from individuals residing in the zip code. The parameters of the model were estimated with the POISSON procedure in STATA/IC 16.

To describe the total population of youth who received testing at UCLA, we conducted a χ^2^ test to compare basic demographic information between confirmed cases to controls. We conducted logistic regression analysis to determine risk ratios for covariates and symptoms. Potential confounding effects were explored for pre-existing conditions, but none were found to change the β by 10%, so only crude regression results are reported. Student *t*-tests were used to determine whether average number of symptoms was associated with a positive SARS-CoV-2 result. Linear regression and correlation coefficients estimated the number of symptoms by age, and βs between the regression lines for cases and controls were analyzed using a *t*-test. We compared the mean Ct value of asymptomatic vs. symptomatic participants using a *t*-test. Ct values were graphed according to days since symptom onset for each positive SARS-CoV-2 PCR test. Samples were categorized according to disease severity. Asymptomatic or pre-symptomatic cases at the time of testing had the *x*-axis set to 0. Statistical analysis was performed using STATA (Version 14.2) and R (Version 4.0.4), using a two-sided hypothesis, with α < 0.05.

## Results

**Figure 1:**
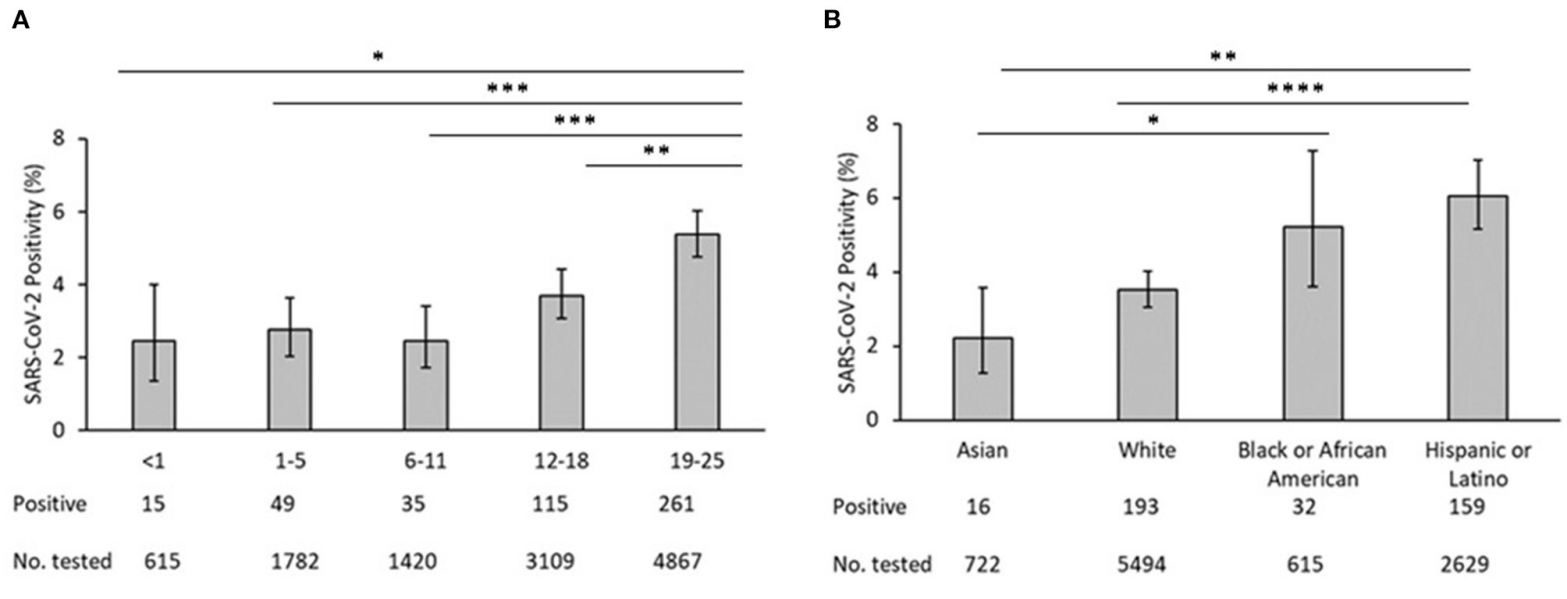
**(A)** SARS-CoV-2 test positivity by age. **(B)** SARS-COV-2 test positivity by race/ethnicity. In each panel, bars represent the mean proportion of positives +/−95% confidence interval. ^*^p < 0.05, ^**^p < 0.01, ^***^p < 0.001, ^****^p < 0.0001.

A total of 27,976 SARS-CoV-2 assays among 11,922 youth aged <25 years were performed during the study period. From this group, 475 youth (4.0%) were identified as confirmed cases while 11,447 youth (96.0%) had only negative test results and were identified as controls. Of 475 confirmed cases, 323 had positive results on RT-PCR and 152 had positive results on IgG enzyme-linked immunosorbent assay (ELISA). Of the 11,922 youth who were tested, the median age was 17.4 (IQR 8.3–21.6); the majority were aged 19–25 years (41.3%) and 12–18 years (26.4%). The positivity rate of 19- to 24-year-olds was significantly higher than in younger age groups (Figure 1A). Differences in positivity among other age groups was not significant. Race/ethnicity was available for 9,460 youth: 58.1% White, 27.8% Hispanic, and 6.5% Black/African American. The positivity rate of Hispanic/Latinx participants was significantly higher than Asians (*p* = 0.002) or White (*p* < 0.0001) but not Black/African-Americans (*p* = 0.79) (Figure 1B). The positivity rate of Asians was significantly lower than that of Blacks/African-Americans (*p* = 0.0229). None of the other pairwise comparisons between races/ethnicities was statistically signficant.

The case–control analysis showed a significant difference in race/ethnicity between cases and controls; cases were more likely to be Hispanic/Latinx, Black/African American, or other and controls were more likely to be White (Table 1). Language spoken at home was significantly different between both groups. Confirmed cases were more likely to have MediCal or safety net insurance as compared to controls, who were more likely to have private insurance. The majority of cases had mild illness (61.3%) with similar proportion of asymptomatic or pre-symptomatic (17.7%) and severe/ critical illness (16.9%). Reason for testing, patient occupation, pets at home, recent travel, and contact with a known or suspected COVID-19 case were associated with a positive result for SARS-CoV-2 (Table 2). Youth who received testing for asymptomatic surveillance were less likely to test positive compared to those who were tested for a known exposure or other reason. Youth with an exposure to suspected or confirmed cases of SARS-CoV-2 were >20 times more likely to test positive for SARS-CoV-2 than those with unknown exposures. Youth with recent travel to high-risk areas were 1.5 times more likely to test positive for SARS-CoV-2 as those without recent travel. Essential worker youth were more likely to test positive for SARS-CoV-2 as compared to students and others. Patients who reported pets at home were less likely to test positive for SARS-CoV-2; however, pet ownership status was unknown in more than half of all patients. Parent occupation, location of visit/ordering provider, and household size were not significantly associated with positive results. Larger household size (>4 members) was more common in cases (14.5%) as compared to controls (8.5%); however, household size was missing for many patients.

**Table 1:** Demographics.

	**SARS-CoV-2 Positive**	**SARS-CoV-2 Negative**	***p*-value**
***n*** **=** **873**	475	398	
**Median age at testing**	19.8 (14.3, 22.4)	19.7 (13.7, 22.4)	0.71
**Age at testing (years)**	***n*** **(%)**	***n*** **(%)**	0.60
<1	14 (2.9)	20 (5.0)	
1–5	49 (10.3)	38 (9.5)	
6–11	35 (7.4)	30 (7.5)	
12–18	78 (16.4)	68 (17.1)	
18–24	299 (62.9)	242 (60.8)	
**Sex**			0.63
Male	239 (50.3)	197 (49.5)	
Female	235 (49.5)	201 (50.5)	
Not specified	1 (0.2)	0 (0.0)	
**Race/ethnicity**			** <0.01**
Asian, American Indian/Alaska Native, Native Hawaiian/Other Pacific Islander	16 (3.4)	35 (8.8)	
Black/African American (Not Hispanic)	29 (6.1)	14 (3.5)	
Hispanic/Latinx	164 (34.5)	88 (22.1)	
White (Not Hispanic)	133 (28.0)	162 (40.7)	
Other/Unknown	133 (28.0)	99 (24.9)	
**Language spoken at home**			** <0.01**
English	389 (81.9)	383 (96.2)	
Non-English	17 (3.6)	3 (0.8)	
Unknown	69 (14.5)	12 (3.0)	
**Type of Insurance**			**0.02**
Medical/Safety Net	107 (22.5)	66 (16.6)	
No Insurance	30 (6.3)	16 (4.0)	
Private Insurance	338 (71.2)	316 (79.4)	
**PMH**			** <0.01**
Obesity (BMI > 98th percentile)	19 (4.2)	1 (0.3)	
Asthma	17 (3.8)	7 (1.8)	
Chronic Pulmonary/Lung Disease	1 (0.2)	0 (0.0)	
Cardiac	11 (2.4)	7 (1.8)	
Immunosuppression	5 (1.1)	4 (1.0)	
Diabetes/pre-diabetes	3 (0.7)	1 (0.3)	
Psych	24 (5.3)	37 (9.5)	
Other	98 (21.6)	161 (41.4)	
None	275 (60.7)	171 (44.0)	
**COVID Outcomes**			
Asymptomatic	84 (17.7)		
Mild	291 (61.3)		
Severe	62 (13.1)		
Critical	18 (3.8)		
Unknown	20 (4.2)		
MIS-C	4 (0.8)		
**Other Outcomes**			
Hospitalized	22 (4.6)		
Hospital LOS (mean, IQR)	3.0 (2.0, 9.0)		
ICU	17 (3.5)		
Mechanical ventilation	4 (0.8)		
Death	1 (0.2)		

*Bold values indicate significance with P < 0.05*.

**Table 2:** Risk factors for SARS-CoV-2 infection.

	**SARS-CoV-2 Positive**	**SARS-CoV-2 Negative**	**OR**	**95% CI**	***p*-value**
	***n* = 475**	***n* = 398**			
	***n* (%)**	***n* (%)**			
**Reason for Testing**					** <0.001**
Exposure	81 (17.1)	48 (12.1)	**REF**	**REF**	
Symptoms	343 (72.2)	139 (34.9)	1.5	(1.0, 2.2)	
Surveillance	46 (9.7)	193 (48.5)	0.1	(0.1, 0.2)	
Other	5 (1.1)	18 (4.5)	0.2	(0.1, 0.4)	
**Location of Visit**					** <0.001**
ED	132 (27.8)	86 (21.6)	**REF**	**REF**	
Outpatient/Telemedicine	293 (61.7)	220 (55.3)	0.9	(0.6, 1.2)	
Other/Unknown	50 (10.5)	92 (23.1)	0.4	(0.2, 0.5)	
**Exposure**					**0.003**
Recent Travel to high-risk area					
No	343 (72.2)	325 (81.7)	**REF**	**REF**	
Yes	69 (14.5)	43 (10.8)	1.5	(1.0, 2.3)	
Unknown	63 (13.3)	30 (7.5)	2.0	(1.3, 3.2)	
**Contact with confirmed/suspected**					** <0.001**
No	55 (11.6)	232 (58.3)	**REF**	**REF**	
Yes	331 (69.7)	65 (16.3)	21.5	(14.6, 32.2)	
Unknown	89 (18.7)	101 (25.4)	3.7	(2.5, 5.6)	
**Occupation (patient)**					**0.002**
Essential Worker	87 (18.3)	39 (9.8)	**REF**	**REF**	
Unemployed	20 (4.2)	19 (4.8)	0.5	(0.2, 1.0)	
Student	250 (52.5)	211 (53.0)	0.5	(0.3, 0.8)	
Other	111 (23.4)	116 (29.1)	0.4	(0.3, 0.7)	
Missing	7 (1.5)	13 (3.3)	0.2	(0.1, 0.6)	
**Occupation (parent)**					0.6898
Essential Worker	42 (8.8)	26 (6.5)	**REF**	**REF**	
Unemployed	5 (1.1)	4 (1.0)	0.8	(0.4, 1.3)	
Student	14 (2.9)	11 (2.8)	0.8	(0.4, 1.1)	
Other	244 (51.4)	220 (55.3)	0.7	(0.3, 2.0)	
Missing	170 (35.8)	137 (34.4)	0.8	(0.2, 3.4)	
**Pets at home**					** <0.001**
No	180 (37.9)	132 (33.2)	**REF**	**REF**	
Yes	7 (1.5)	37 (9.3)	0.1	(0.1, 0.3)	
Unknown	288 (60.6)	229 (57.5)	0.9	(0.7, 1.2)	
Household Size^*^					** <0.001**
>4	69 (14.5)	34 (8.5)	**REF**	**REF**	
1–2	40 (8.4)	11(2.8)	1.8	(0.8, 4.1)	
3–4	169 (35.6)	99 (24.9)	0.8	(0.5, 1.4)	
Missing	197 (41.5)	254 (63.8)	0.4	(0.2, 0.6)	

*OR, odds ratio; CI, confidence interval*.

* *including patient. Bold values indicate significance with P < 0.05*.

**Figure 2:**
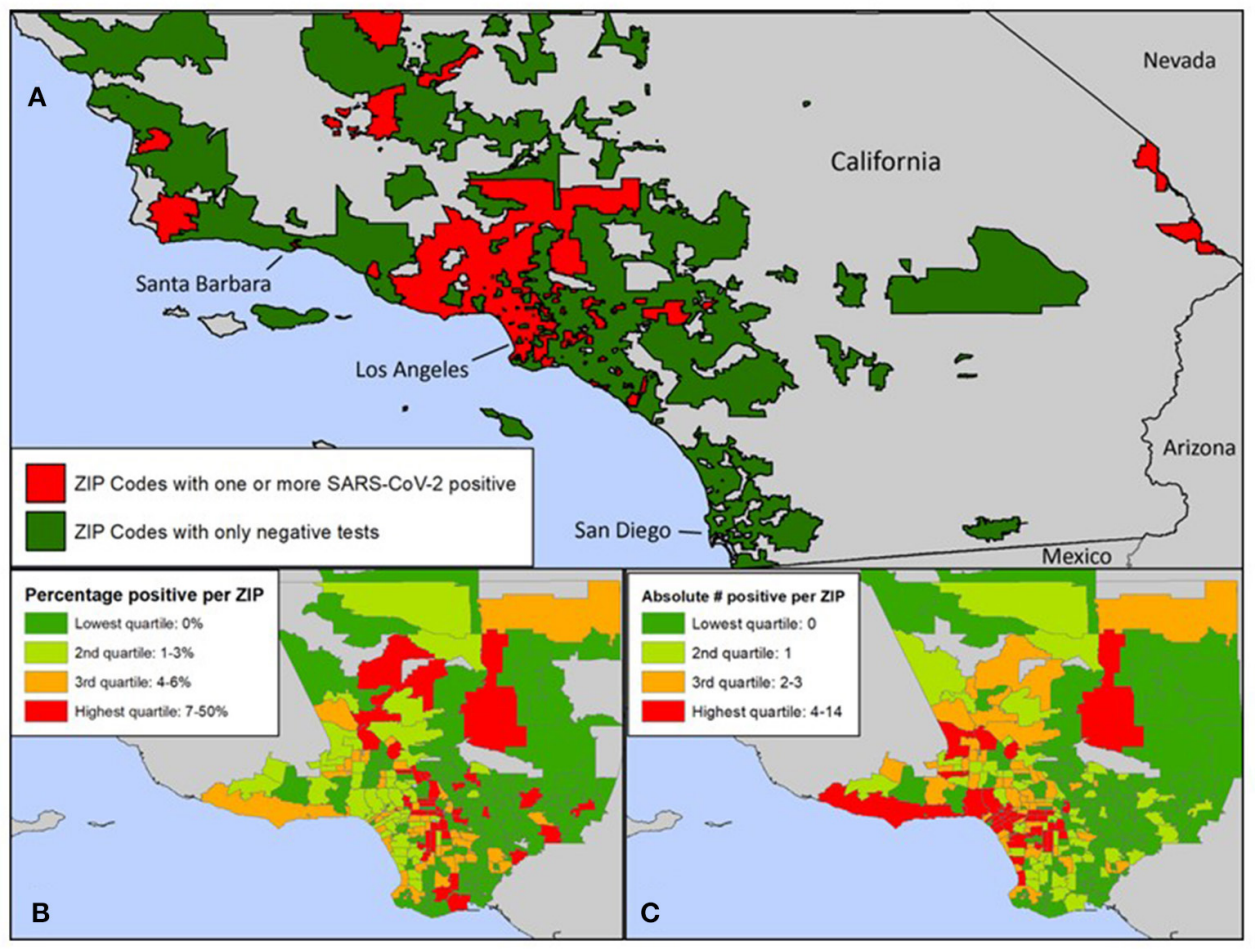
**(A)** Zip codes in Southern California with SARS-CoV-2 tests at UCLA. **(B)** Zip codes within Los Angeles County with positive SARS-CoV-2 tests at UCLA: Percentage of positive tests. **(C)** Zip codes within Los Angeles County with positive SARS-CoV-2 tests at UCLA: Absolute number of positive tests. **(A)** Zip codes in green reflect residents who were tested at UCLA and were negative. Zip codes in red reflect residents who were tested at UCLA and were positive. **(A–C)** Zip codes of youth tested for SARS-CoV-2 at UCLA included zip codes in Los Angeles, Santa Barbara, Ventura, San Bernardino, Riverside, San Diego, and Orange Counties. The number of cases of SARS-CoV-2 positive youth under 25 per zip code was significantly associated with the proportion of residents who identified as Hispanic/Latinx.

Zip code was statistically associated with SARS-CoV-2 infection. Zip codes with a higher proportion of Hispanic/Latinx residents and residents living under the poverty line had higher number of SARS-CoV-2 cases (Table 3). Figure 2A describes the geographical spread of all youth SARS-CoV-2 results across southern California with zip code information available (*n* = 277); 132 (47.7%) had positive SARS-CoV-2 results. Figures 2B,C focus on zip codes within Los Angeles County where most of the patients resided. These panels show that while many zip codes on the west side of Los Angeles (close to UCLA) had higher absolute number of positive tests (highest quartile), the percent positivity was not as high (second and third quartiles) due to the high number of tests performed. Conversely, three zip codes on the east side of Los Angeles had high test positivity (top quartile), but low absolute numbers of positive tests (2nd quartile). There were several zip codes in the 3rd and 4th quartiles for both test positivity and absolute numbers of positive tests. These zip codes concentrated in central and south Los Angeles as well as Palmdale and Santa Clarita areas.

**Table 3:** Effect of demographic variables on the number of SARS-CoV-2 cases in youth <25 per ZIP Code in Los Angeles County.

**Effect**	**Incidence Rate Ratio (95% CI)**	***p***
Proportion Asian	1.15 (0.11–11.65)	0.905
Proportion Black/African American	2.05 (0.28–15.12)	0.48
Proportion Hispanic/Latinx	3.54 (1.57–7.99)	0.002
Proportion White	0.833 (0.16–4.39)	0.83

*CI, confidence interval*.

**Figure 3:**
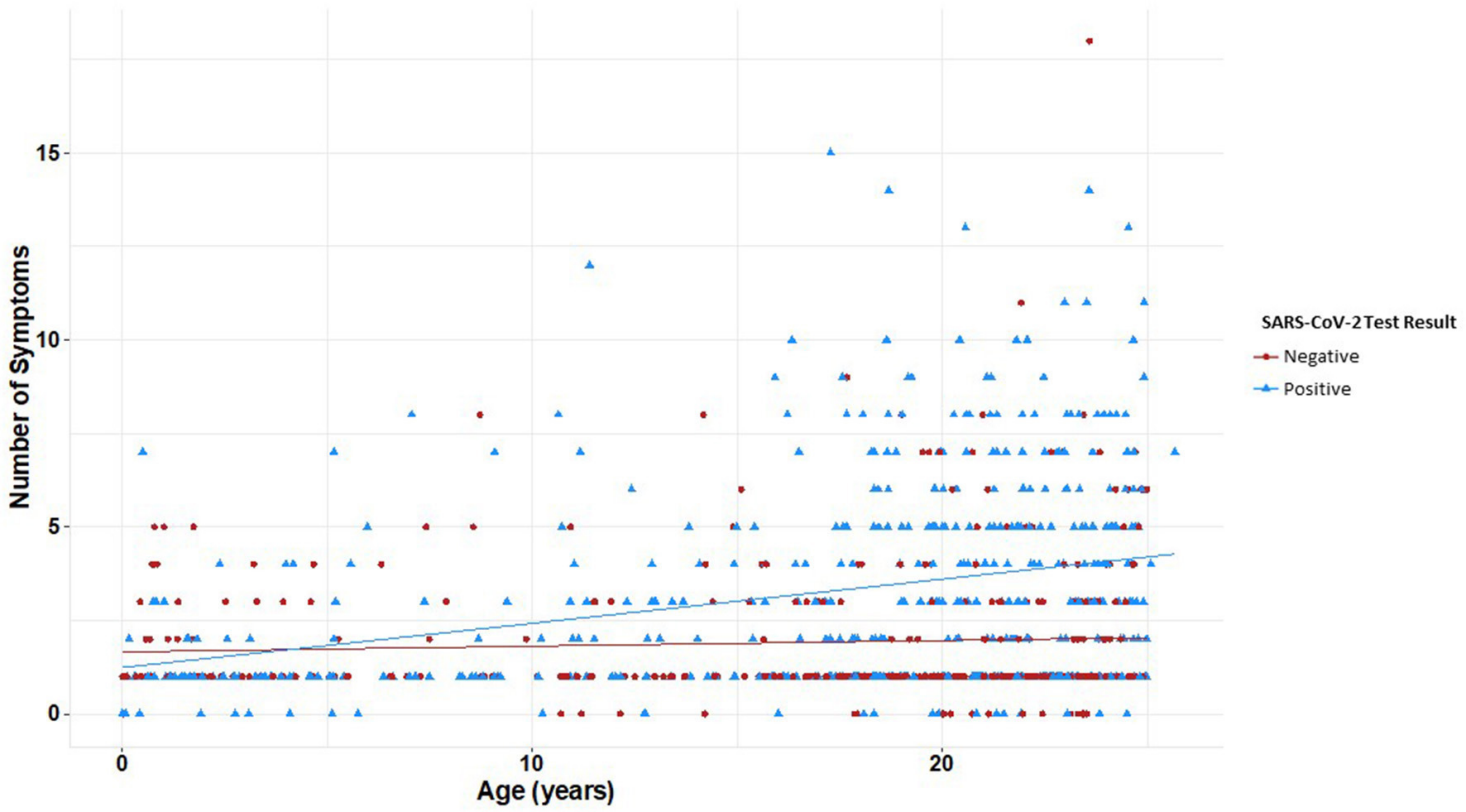
Number of reported symptoms at time of SARS-CoV-2 testing by age and test result. Linear regression and correlation coefficients estimate the change in number of symptoms by age. Youth with positive SARS-CoV-2 results had a significant correlation between age and number of symptoms (*r* = 0.30, *p* < 0.001); youth with negative results had no significant correlation between age and number of symptoms (*r* = 0.06, *p* = 0.25). Presence of symptoms increased significantly with age (*p* < 0.001). Youth with positive SARS-CoV-2 results are plotted in blue and youth with negative SARS-CoV-2 results are in red.

All symptoms were associated with a positive SARS-CoV-2 test (*p* <0.05) except congestion/runny nose, headache, and other neurological (altered consciousness, seizure, inability to walk, fainting, and dizziness) and dermatologic findings (skin rashes/ulcers) (Table 4). Youth with no symptoms at the time of testing were 0.74 times as likely to test positive for SARS-CoV-2 than youth with symptoms (OR 0.7, 95% CI 0.7–0.8). The average number of symptoms reported at the testing visit was significantly different between both groups (*p* <0.05). When further stratified by age, patterns of symptom presentation varied significantly by age group with more symptoms becoming associated with positive tests in older age subsets (Table 5). Among participants with positive SARS-CoV-2 results, there was a moderate and significant positive correlation between age and number of symptoms (*r* = 0.30, *p* <0.001); presence of symptoms increased significantly with age (Figure 3). Among patients with negative results, however, there was no significant association between number of symptoms and age.

**Table 4:** Symptoms at time of SARS-CoV-2 testing.

	**SARS-CoV-2 Positive**	**SARS-CoV-2 Negative**	**OR**	**95% CI**

	***n*** **=** **475**	***n*** **=** **398**		
	***n*** **(%)**	***n*** **(%)**		
Asymptomatic/Pre-symptomatic	131 (27.6)	229 (57.75)	0.7	**(0.7, 0.8)**
**Respiratory**				
Cough/Sneezing	172 (36.2)	72(18.1)	1.3	**(1.2, 1.3)**
Congestion/Runny Nose	84 (17.7)	59 (14.8)	1.1	(1.0, 1.2)
Shortness of Breath/Wheezing	56 (11.8)	18 (4.5)	1.3	**(1.1, 1.4)**
Chest Pain	32 (6.7)	6 (1.5)	1.4	**(1.2, 1.6)**
Sore Throat	129 (27.2)	66 (16.6)	1.2	**(1.1, 1.3)**
Other^a^	5 (1.1)	5 (1.3)	1.0	(0.7, 1.3)
**Gastrointestinal**				
Diarrhea	45 (9.5)	23 (5.8)	1.1	**(1.0, 1.3)**
Vomiting	43 (9.1)	16 (4.0)	1.2	**(1.1, 1.4)**
Other^b^	39 (8.2)	18 (4.5)	1.2	**(1.0, 1.3)**
**Constitutional**				
Fever/Chills	171 (36.0)	66 (16.6)	1.3	**(1.2, 1.4)**
Myalgia/Joint pain	111 (23.4)	22 (5.5)	1.4	**(1.3, 1.5)**
Fatigue	105 (22.1)	51 (12.8)	1.2	**(1.1, 1.3)**
**Neurological**				
Loss of Taste/Smell	72 (15.2)	4 (1.0)	1.6	**(1.4, 1.7)**
Headache	89 (18.7)	24 (6.0)	1.3	**(1.2, 1.5)**
Other^c^	16 (3.4)	9 (2.3)	1.1	(0.9, 1.3)
**Dermatological**	10 (2.1)	7 (1.8)	1.0	(0.8, 1.3)
Other*^d^	119 (25.1)	32 (8.0)	1.3	**(1.2, 1.5)**
Average # Symptoms (mean, sd)	3.3 (2.8)	1.9 (1.9)	1.1	**(1.0, 1.1)^e^**

*OR, odds ratio; CI, confidence interval*.

a *Respiratory “Other” includes ear pain, conjunctivitis*.

b *Gastrointestinal “Other” includes abdominal pain, constipation or change in bowl habits, lack of app, bloating*.

c *Neurological “Other” includes altered consciousness, seizures, unable to walk, fainting, dizziness, agitation*.

d *“Other” includes lymphadenopathy, swelling/edema*.

e *Confidence interval for Student's t-test*.

*Boldface values were significant with p < 0.05*.

**Table 5:** Symptoms at time of SARS-CoV-2 testing stratified by age.

	**SARS-CoV-2 Positive**	**SARS-CoV-2 Negative**	**OR**	**95% CI**

	***n*** **(%)**	***n*** **(%)**		
**Under 6 years (*****n*** **=** **121)**
Asymptomatic/Pre-symptomatic	24 (38.1)	34 (58.6)	0.8	**(0.7, 1.0)**
Respiratory	16 (25.4)	18 (31.0)	0.9	(0.8, 1.1)
Gastrointestinal	5 (7.9)	7 (12.1)	0.9	(0.7, 1.2)
Constitutional	22 (34.9)	19 (32.8)	1.0	(0.8, 1.2)
Neurological	2 (3.2)	2 (3.4)	1.0	(0.6, 1.6)
Dermatological	2 (3.2)	3 (5.2%)	0.9	(0.6, 1.4)
Other	15 (23.8)	1 (1.7)	1.6	**(1.3, 2.1)**
Average # Symptoms (mean, sd)	1.6 (1.5)	1.8 (1.3)	1.0	(0.9, 1.0)
**6–11 years (*****n*** **=** **65)**
Asymptomatic/Pre-symptomatic	18 (51.4)	18 (60.0)	0.9	(0.7, 1.2)
Respiratory	11 (31.4)	10 (33.3)	1.0	(0.8, 1.3)
Gastrointestinal	8 (22.9)	5 (16.7)	1.1	(0.8, 1.5)
Constitutional	8 (22.9)	7 (23.3)	1.0	(0.7, 1.3)
Neurological	8 (22.9)	2 (6.7)	1.4	(1.0, 1.9)
Dermatological	2 (5.7)	1 (3.3)	1.1	(0.6, 2.1)
Other	6 (17.1)	1 (3.3)	1.4	(1.0, 2.1)
Average # Symptoms (mean, sd)	2.6 (2.7)	2 (1.8)	1.0	(1.0, 1.1)
**12–17 years (*****n*** **=** **146)**
Asymptomatic/Pre-symptomatic	26 (33.3)	41 (60.3)	0.8	**(0.7, 0.9)**
Respiratory	46 (59.0)	15 (22.1)	1.5	**(1.3, 1.7)**
Gastrointestinal	11 (14.1)	5 (7.4)	1.2	(0.9, 1.5)
Constitutional	33 (42.3)	10 (14.7)	1.4	**(1.2, 1.7)**
Neurological	18 (23.1)	5 (7.4)	1.3	(1.1, 1.7)
Dermatological	2 (2.6)	1 (1.5)	1.1	(0.6, 2.0)
Other	11 (14.1)	8 (11.8)	1.1	(0.8, 1.3)
Average # Symptoms (mean, sd)	2.9 (2.6)	1.7 (1.7)	1.1	**(1.0, 1.1)**
**18 years and older (*****n*** **=** **541)**
Asymptomatic/Pre-symptomatic	63 (21.1)	135 (55.8)	0.7	**(0.6, 0.7)**
Respiratory	174 (58.2)	66 (27.3)	1.4	**(1.3, 1.5)**
Gastrointestinal	66 (22.1)	20 (8.3)	1.3	**(1.2, 1.4)**
Constitutional	168 (56.2)	54 (22.3)	1.4	**(1.3, 1.5)**
Neurological	118 (39.5)	24 (9.9)	1.5	**(1.3, 1.6)**
Dermatological	4 (1.3)	2 (0.8)	1.1	(0.8, 1.7)
Other	87 (29.1)	22 (9.1)	1.4	**(1.2, 1.5)**
Average # Symptoms (mean, sd)	3.8 (2.9)	2 (2.1)	1.1	**(1.0, 1.1)**

*OR, odds ratio; CI, confidence interval*.

*Boldface values were significant with p < 0.05*.

**Figure 4:**
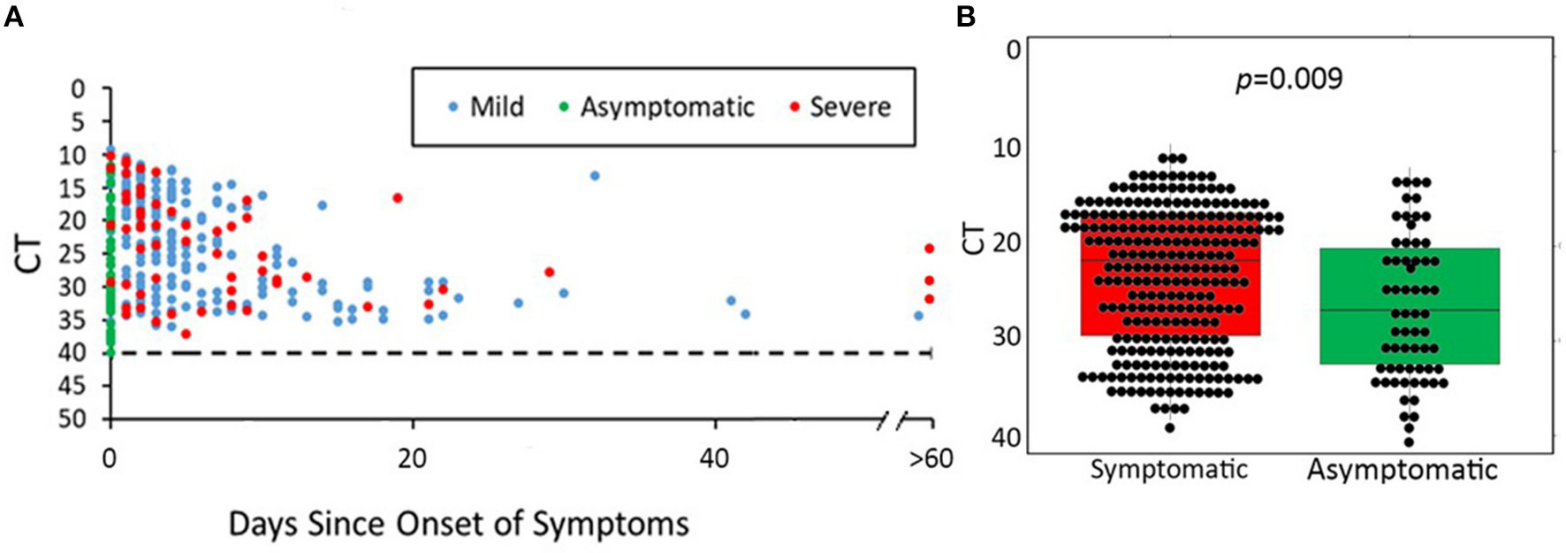
SARS-CoV-2 PCR cycle threshold (Ct) for available nasopharyngeal specimens (NP) from 475 youth with COVID-19. **(A)** Days of symptoms vs. cycle threshold (Ct) according to COVID-19 severity. **(B)** Median Ct values of positive PCR results in symptomatic vs. asymptomatic youth. **(A)** shows SARS-CoV-2 Ct values according to days since symptom onset for each positive SARS-CoV-2 PCR test. Asymptomatic/pre-symptomatic cases were distributed along the entire height of the *y*-axis from Ct values of 10 (representing the highest viral load) to Ct values of 40 (negative cutoff for a positive RT-PCR result). Mild/moderate cases represented the majority of data points (*n* = 213) and demonstrated a wide range of Ct values (10–40). Severe cases were fewer in number (*n* = 59) and generally had lower Ct values. **(B)** Median PCR Ct values of asymptomatic youth were significantly lower than that of symptomatic youth (21.5 vs. 26.7, *p* = 0.009).

**Figure 5:**
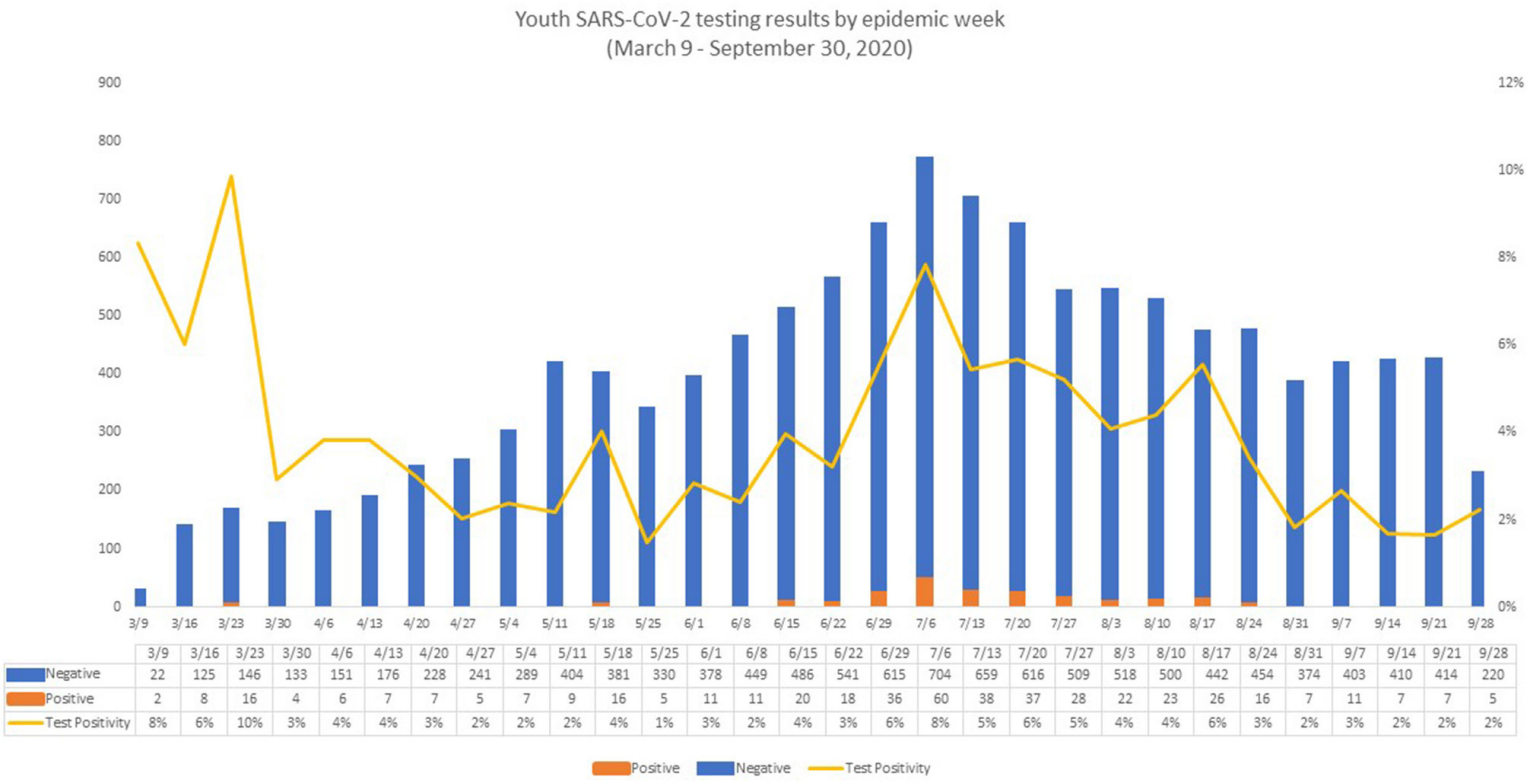
Youth SARS-CoV-2 testing results by epidemic week. The figure reflects the evolution of the pandemic in Los Angeles County, with very few positive tests in the first week (two positive SARS-CoV-2 PCR tests) and in the last week of the study period (five positive SARS-CoV-2 PCRs). The number of positive SARS-CoV-2 PCR tests peaked in week 18 (July 6, 2020 to July 12, 2020), corresponding roughly with the summer peak of COVID-19 in Los Angeles County (23, 24). During week 18, the number of both positive and negative SARS-CoV-2 PCR tests reached the highest number during our observation period, with 60 positive SARS-CoV-2 PCRs and 704 negative SARS-CoV-2 PCRs, resulting in a positivity rate of 7.8%. Week 1 also showed a high positivity rate (6.7%), but a low number of tests were performed (*n* = 30) with the lowest number of positive tests (*n* = 2) observed that week. After the Week 18 peak, the number of positive tests remained elevated (22–37 positive tests/week) until week 25 (August 24, 2020 to August 30, 2020). At that point, the number of positive results began to decline.

Ct values were evaluated for 328 positive RT-PCRs, representing 328 unique cases (Figure 4). The majority of cases were detected <20 days since symptom onset; however, two severe cases remained with a Ct value of <25 for more than 60 days following the onset of symptoms. The median Ct value for symptomatic cases was 21.5 compared to 26.7 for asymptomatic cases. Symptomatic youth had lower median Ct values than those who were asymptomatic (*t* = 3.189, df = 91.764, *p* = 0.0009, Figure 4B). In a direct comparison of mild/moderate cases vs. severe, there was no statistically significant difference in mean Ct values between groups (*p* = 0.95). Figure 5 shows the number of positive and negative SARS-CoV-2 PCR tests done each week during the study period, which encompassed the entire first wave of the pandemic in Southern California.

## Discussion

In our youth population, geographic predictors of SARS-CoV-2 infection were consistent with inequities that have been well-established in the general population throughout the COVID-19 pandemic (10, 11). Markers of inequity were associated with infection similar to adults (11, 12, 25). Epidemiologic and clinical predictors of SARS-CoV-2 infection were also identified. In both our larger cohort and case–control analysis, older youth displayed infection rates and symptoms more similar to the adult population than younger peers. Epidemiologic factors such as travel, exposure, and past medical history were also predictive of SARS-CoV-2 infection in youth. A lack of association between viral load and disease severity was noted; nevertheless, asymptomatic youth had significantly lower virus loads as compared to symptomatic participants, which likely reflects important implications for transmission.

The geographic findings in our young participants were consistent with the current literature on COVID-19 in adults, particularly with a Los Angeles County ecological study that reported higher crude rates of SARS-CoV-2 positivity among Latinx adult residents and people living below the poverty line (10). The link between neighborhood and higher infection rates has been well-established and points to structural inequalities including poor access to preventive health measures and medical care (11). Both our cohort and case–control analyses demonstrated a racial breakdown of SARS-CoV-2 infection in youth consistent with what has been previously reported in the literature (7, 13, 14).

Socioeconomic factors continue to be an important driver of the COVID-19 pandemic, highlighting inequities in our society and providing an important area for future work. There has been concern for the impact of COVID-19 on immigrant communities; however, there is little known about the association between immigration status and COVID-19 infection (12, 23). Non-English language spoken at home was used as an indicator of immigrant households and was associated with increased risk for SARS-CoV-2 infection. This has concerning implications given that households in which English is not the primary language may not have access to information about how to reduce potential exposures, enhance protection against the virus, and access vaccines.

The majority of youth tested at our medical center were 19–25 years of age, with a higher positivity rate observed in this age group, consistent with trends in COVID-19 testing (2). Older children, 12 years of age and above, behaved more like adult cohorts in terms of symptom presentation. Pediatric studies showed that children typically present with mild or asymptomatic infection, whereas adults have a wider range of symptoms, in addition to being at higher risk for disease (2, 4, 5, 26). An increasing number of symptoms at the time of testing was noted with older age in our cohort. This finding has important implications for re-opening of junior high, high schools, and universities, where transmission dynamics may be more similar to that of the general community than that observed in younger children in pre-school and elementary schools (27).

For youth in our study, the patient's occupation was a predictor of positive SARS-CoV-2 results, while parental occupation was not significantly associated with infection. This likely reflects the age, socioeconomic, and racial distribution of our cohort. Youth working in essential jobs would not have been able to follow stay-at-home orders or socially distance due to their jobs. Similarly, racial minority groups are more likely to be employed in essential occupations (25). Despite concerns early in the pandemic for transmission *via* household pets (28), our study found that having pets at home was associated with lower risk for SARS-CoV-2 infection. Although the mechanism underlying this association is not clear and there may be concern that data are not missing at random, pets at home could be indicators of ability to comply with stay-at-home orders and social distancing rules, thus a proxy for socioeconomic status or other confounder.

We identified clinical predictors of SARS-CoV-2 infection that can be used to guide SARS-CoV-2 testing in youth. Exposure to known or suspected SARS-CoV-2 infection was predictive of SARS-CoV-2 infection in our study and has been established as a risk factor for SARS-CoV-2 infection in studies in adults (6). However, symptoms in our study were more frequently associated with SARS-CoV-2 infection than in prior reports. In one large study of pediatric patients, only fever and chills were predictive of a positive test (4). This study, however, was limited to patients tested due to symptoms or exposure to SARS-CoV-2. Our study, conversely, included youth who were tested for any reason, including asymptomatic surveillance. Consequently, a large proportion of asymptomatic youth were included, which contributed to the likelihood of symptoms being associated with positive results. In this sense, our study captured a population that was more similar to the general population of youth with low prevalence of symptomatic COVID-19, rendering our results more generalizable (13). Our findings suggest that symptom-driven testing may be appropriate in youth.

Viral load, as represented by cycle threshold, did not vary with symptom severity if symptoms were indeed present, which is consistent with the findings of pediatric studies (3). However, this is distinct from studies in adults, where SARS-CoV-2 viral load has been shown to be associated with disease severity (17, 29). Additionally, viral load was higher in children with symptoms as compared to those without symptoms in the present study, one pediatric study, and a mixed adult and pediatric study (16, 18). Our findings differ from several studies in both adult and pediatric populations that reported no difference in viral load among patients with asymptomatic vs. symptomatic disease (7, 15, 17). Our results demonstrate that lower viral replication is present in asymptomatic disease, which has important implications for viral transmission, i.e., lower risk of transmission from asymptomatic youth. Nevertheless, it is difficult to determine where asymptomatic youth were in their course of illness at the time of testing. Almost 240 of our participants were tested for asymptomatic surveillance purposes (pre-procedure, hospital admission or for school or travel), accounting for 50.5% of the case–control study population.

Our study has strengths and limitations. We were limited to testing done within our own health system and, as such, could not account for untested youth with SARS-CoV-2 infection and those tested outside of our network. Pediatric patients admitted to our academic medical center tended to reflect the high complexity of a tertiary care children's hospital. Because hospitalized patients were screened for SARS-CoV-2 on admission and pre-procedure and pediatric patients requiring hospitalization at our institution are often chronically ill, this likely skewed our overall study population, particularly controls with negative RT-PCRs to a medically complex group of youth. Furthermore, our study includes youth tested from March to September 2020, the first wave of the COVID-19 pandemic in Southern California, and the epidemiologic profile of SARS-CoV-2 infection in youth may have changed, especially in the context of new variants and lockdowns. Further studies are warranted to examine these questions in later waves. As testing volume in pediatric patients remains lower than in adults (2), this could contribute to older age groups being more often represented in our patient population. Nevertheless, we collected more detailed data on a larger number of patients than has been reported to date. Furthermore, we were able to map SARS-CoV-2 infections in children and youth along a very large metropolitan area of Los Angeles County and demonstrate important associations with demographic parameters also for pediatric patients.

## Conclusion

In summary, we identified social and epidemiologic predictors of SARS-CoV-2 infection in a large youth population followed at an urban academic medical center and associated clinics. Our findings highlight the ways in which structural racism has resulted in disproportionate rates of infection of SARS-CoV-2 in children and youth from communities of color and certain geographic regions. Finding ways to improve the ability of these communities to prevent infection, through vaccination and appropriate mitigation policies at schools and workplaces, is crucial for successful containment of the pandemic. Because older youth are more similar to adults in regard to SARS-CoV-2 infection, it is important to tailor policies of school reopening to elementary, middle, high schools, and universities differently. Symptom and exposure-based screening policies can be effective especially in older youth population brackets.

## Data Availability Statement

The raw data supporting the conclusions of this article will be made available by the authors, without undue reservation.

## Ethics Statement

The studies involving human participants were reviewed and approved by UCLA Institutional Review Board. Written informed consent from the participants' legal guardian/next of kin was not required to participate in this study in accordance with the national legislation and the institutional requirements.

## Author Contributions

CN conceptualized and designed the study, designed the data collection instruments, interpreted the data, drafted the initial manuscript, reviewed, and revised the manuscript. KN-S conceptualized and designed the study, interpreted the data, reviewed, and revised the manuscript. TS collected data, interpreted the data, reviewed, and revised the manuscript. TF, TK, and ES analyzed the data, interpreted the data, reviewed, and revised the manuscript. MC contributed to the design of the study, interpreted the data, reviewed, and revised the manuscript. OG, SC, CL, MT, and WS collected data and critically reviewed the manuscript for important intellectual content. All authors contributed to the article and approved the submitted version.

## Funding

MC received funding from the T32 Post-Doctoral Training Program Grant (T32MH080634, PIs Currier and Gorbach). ES was supported by the UCLA South American Program in HIV Prevention Research Program, NIH/NIMH (R25 MH087222, PI: J Clark). TF, TK, and KN-S received support from the NIH/NIAID (AI140718, PI Nielsen). KN-S received support from the UCLA W. M. Keck Foundation COVID 19 Research Award Program.

## Conflict of Interest

The authors declare that the research was conducted in the absence of any commercial or financial relationships that could be construed as a potential conflict of interest.

## Publisher's Note

All claims expressed in this article are solely those of the authors and do not necessarily represent those of their affiliated organizations, or those of the publisher, the editors and the reviewers. Any product that may be evaluated in this article, or claim that may be made by its manufacturer, is not guaranteed or endorsed by the publisher.

## Abbreviations

SARS-CoV-2: severe acute respiratory syndrome coronavirus-2
COVID-19: Coronavirus-associated disease 2019
UCLA, University of California: Los Angeles
Ct: cycle threshold
IRB: Institutional Review Board
PCR: polymerase chain reaction
RT-PCR, reverse-transcriptase polymerase chain reaction, ELISA: enzyme-linked immunosorbent assay
MIS-C: multi-system inflammatory syndrome in children
OR: odds ratio
CI: confidence interval.
